# Role of detrusor PDGFRα^+^ cells in mouse model of cyclophosphamide-induced detrusor overactivity

**DOI:** 10.1038/s41598-022-09155-3

**Published:** 2022-03-24

**Authors:** Haeyeong Lee, Byoung H. Koh, Lauren E. Peri, Holly J. Woodward, Brian A. Perrino, Kenton M. Sanders, Sang Don Koh

**Affiliations:** 1 Department of Physiology and Cell Biology, School of Medicine, University of Nevada, Reno, NV 89557 USA; 2 The Roslin Institute, The University of Edinburgh, Easter Bush Campus, Midlothian, EH25 9RG UK

**Keywords:** Physiology, Urology

## Abstract

Cyclophosphamide (CYP)-induced cystitis is a rodent model that shares many features common to the cystitis occurring in patients, including detrusor overactivity (DO). Platelet-derived growth factor receptor alpha positive (PDGFRα^+^) cells have been proposed to regulate muscle excitability in murine bladders during filling. PDGFRα^+^ cells express small conductance Ca^2+^-activated K^+^ channels (predominantly SK3) that provide stabilization of membrane potential during filling. We hypothesized that down-regulation of the regulatory functions of PDGFRα^+^ cells and/or loss of PDGFRα^+^ cells generates the DO in CYP-treated mice. After CYP treatment, transcripts of *Pdgfrα* and *Kcnn3* and PDGFRα and SK3 protein were reduced in detrusor muscle extracts. The distribution of PDGFRα^+^ cells was also reduced. Inflammatory markers were increased in CYP-treated detrusor muscles. An SK channel agonist, CyPPA, increased outward current and hyperpolarization in PDGFRα^+^ cells. This response was significantly depressed in PDGFRα^+^ cells from CYP-treated bladders. Contractile experiments and ex vivo cystometry showed increased spontaneous contractions and transient contractions, respectively in CYP-treated bladders with a reduction of apamin sensitivity, that could be attributable to the reduction in the SK conductance expressed by PDGFRα^+^ cells. In summary, PDGFRα^+^ cells were reduced and the SK3 conductance was downregulated in CYP-treated bladders. These changes are consistent with the development of DO after CYP treatment.

## Introduction

Interstitial cystitis (IC) is characterized by suprapubic and/or bladder pain that is accompanied with an increase in urinary urgency, frequency and nocturia^1,2^. The pathology of IC does not follow that of other diseases/syndromes in the bladder, such as carcinoma, urinary tract infections or cystitis introduced by radiation or medication^3,4^. Although the pathogenesis of IC is still not fully understood, several factors have been suggested which include neuronal, urothelium, and myogenic causes with likely associated inflammation^4^.

Cyclophosphamide (CYP) causes cystitis in humans^5,6^. One of the commonly used models for cystitis in rodents is CYP-induced cystitis, usually induced by injections^7^. This model shares many features with cystitis occurring in human patients treated with CYP, as well as common features with bladder pain syndrome/interstitial cystitis (BPS/IC)^8–11^. CYP causes functional and histological changes in humans and rodents^12,13^, such as expression of receptors and signaling molecules in the urothelium/mucosa. CYP-induced changes include up-regulation of nitric oxide synthase^10,14,15^, increased urothelial muscarinic M5 receptors^16^, and mucosal permeability^15^. Furthermore, after CYP-injection, smaller micturition volumes have been noted^17–19^ that likely are due to upregulated afferent and efferent neural effects^16,20–22^. CYP can also induce detrusor overactivity (DO)^23^. CYP-treated mice showed upregulation of connexin 43 (GJA1) and gap junction blockers attenuated spontaneous contractions in CYP-treated strips, and decreased urinary frequency with increased total voided volume in voiding behavior test of CYP-treated bladder^24^. However, the functional changes in the detrusor interstitial cells responsible for DO have not been determined.

Recently, a unique population of interstitial cells were identified in the bladder. These cells are immunopositive for platelet-derived growth factor receptor alpha (PDGFRα), and therefore are referred to by this chemical coding, i.e. PDGFRα^+^ cells. PDGFRα^+^ cells have been identified in murine, pig and human detrusor muscles^25,26^. These cells are located on the edges of smooth muscle bundles and have multiple branches that may couple to and form an electrical syncytium with smooth muscle cells (SMCs)^25,27,28^. The function of PDGFRα^+^ cells in detrusor muscles has been investigated with molecular and electrophysiological techniques. PDGFRα^+^ cells displayed higher current density of small conductance Ca^2+^-activated K^+^ (SK) channels^26,29^, as compared to detrusor SMCs. Activation of SK channels in PDGFRα^+^ cells could provide a stabilizing influence on smooth muscle excitatiiby. We hypothesized that loss-of-function in PDGFRα^+^ cells or SK channels could lead to DO in cystitis. This study describes investigation into the molecular and functional changes that occur in detrusor PDGFRα^+^ cells and how these changes relate to increased detrusor overactivity in CYP-injected bladder.

## Results

### Downregulation of PDGFRα and SK channel in murine bladders from CYP-induced cystitis

**Figure 1:**
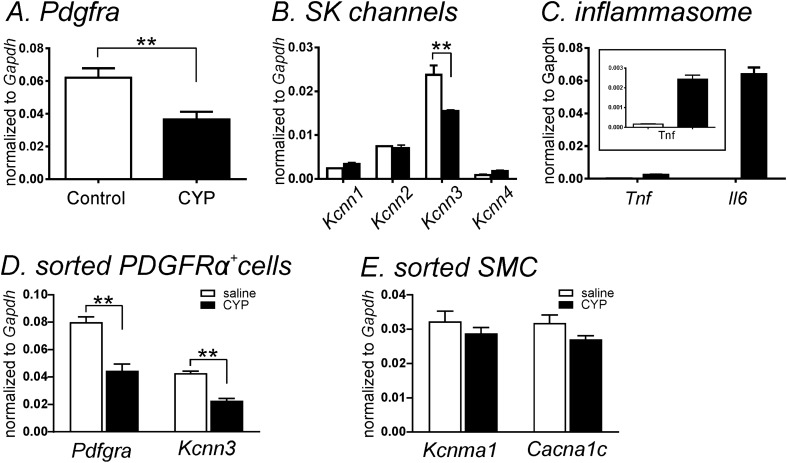
Quantitative analysis of transcripts in control and CYP-treated detrusor muscles. (**A**) Transcripts for *Pdgfra* in control and CYP-treated detrusor muscle. (**B**) Transcripts for *Kcnn* in control and CYP-treated detrusor muscle. (**C**) Transcripts for *Tnf* and *Il6* in control and CYP-treated detrusor muscle. (**D**) Comparison of transcriptional expression of *Pdgfra* and *Kcnn3* from sorted PDGFRα^+^ cells in saline-injected (white bar) and CYP-treated detrusors. (**E**) Comparison of transcriptional expression of *Kcnma1*and *Cacna1c* from sorted smooth muscle cells (SMC) in saline-injected (white bar) and CYP-treated detrusors. Expression of all transcripts was normalized to *Gapdh*. **Denotes *P* < 0.01 by unpaired t-test (n = 4 in all samples).

We compared the transcriptional expression of *Pdgfra and Kcnn1*–*4* in detrusor muscles from CYP-treated and saline-treated mice. *Pdgfra* and *Kcnn3* (SK3) were significantly downregulated (*P* < 0.01 by unpaired t-test), but neither SK1–2 (*Kcnn1 and Kcnn2*) nor IK (*Kcnn4*) were changed significantly after CYP-treatment (n = 4, Fig. 1A,B). Detrusor muscles from CYP-treated mice displayed a significant increase in *Il6* and *Tnf* (inflammatory markers) (n = 4, Fig. 1C) suggesting the onset of bladder inflammation after CYP treatment. We further examined the transcriptional expression of *Pdgfra* and *Kcnn3* in sorted PDGFRα^+^ cells from saline- and CYP-treated PDGFRα/eGFP mice (see “Methods” section). *Pdgfra* and *Kcnn3* were significantly decreased in CYP-treated mice in comparison to mice treated with saline (n = 4, *P* < 0.01 in both genes, Fig. 1D). Transcriptional changes were also evaluated in detrusor smooth muscle cells (SMC). SMCs were isolated from saline- and CYP-treated smMHC/Cre/eGFP (see “Methods” section) mice, as previously described^26^. Main excitability-related genes in detrusor SMC, *Kcnma1* (BK channels) and *Cacna1c* (L-type Ca^2+^ channels) were unchanged in CYP-treated SMC (n = 4, Fig. 1E).

**Figure 2:**
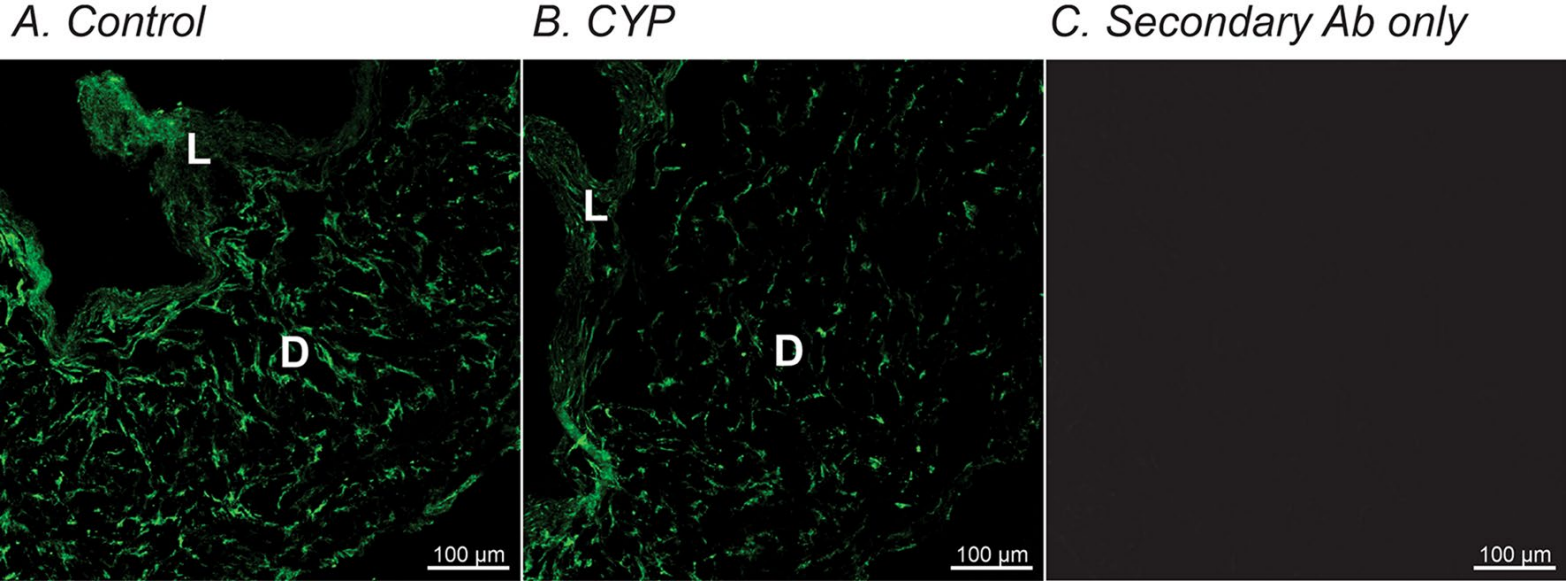
Immunohistochemistry of PDGFRα in murine detrusor from control and CYP-treated bladders. (**A**) Whole mount detrusor with PDGFRα staining (green) from saline-treated control mouse. (**B**) Immunoreactivity with PDGFRα (green) antibody was decreased in CYP-treated bladder detrusor compared with control (**A**). (**C**) Secondary antibody only (no primary antibody control) produce little to no visual background. L and D denote lamina propria and detrusor muscle, respectively.

**Figure 3:**
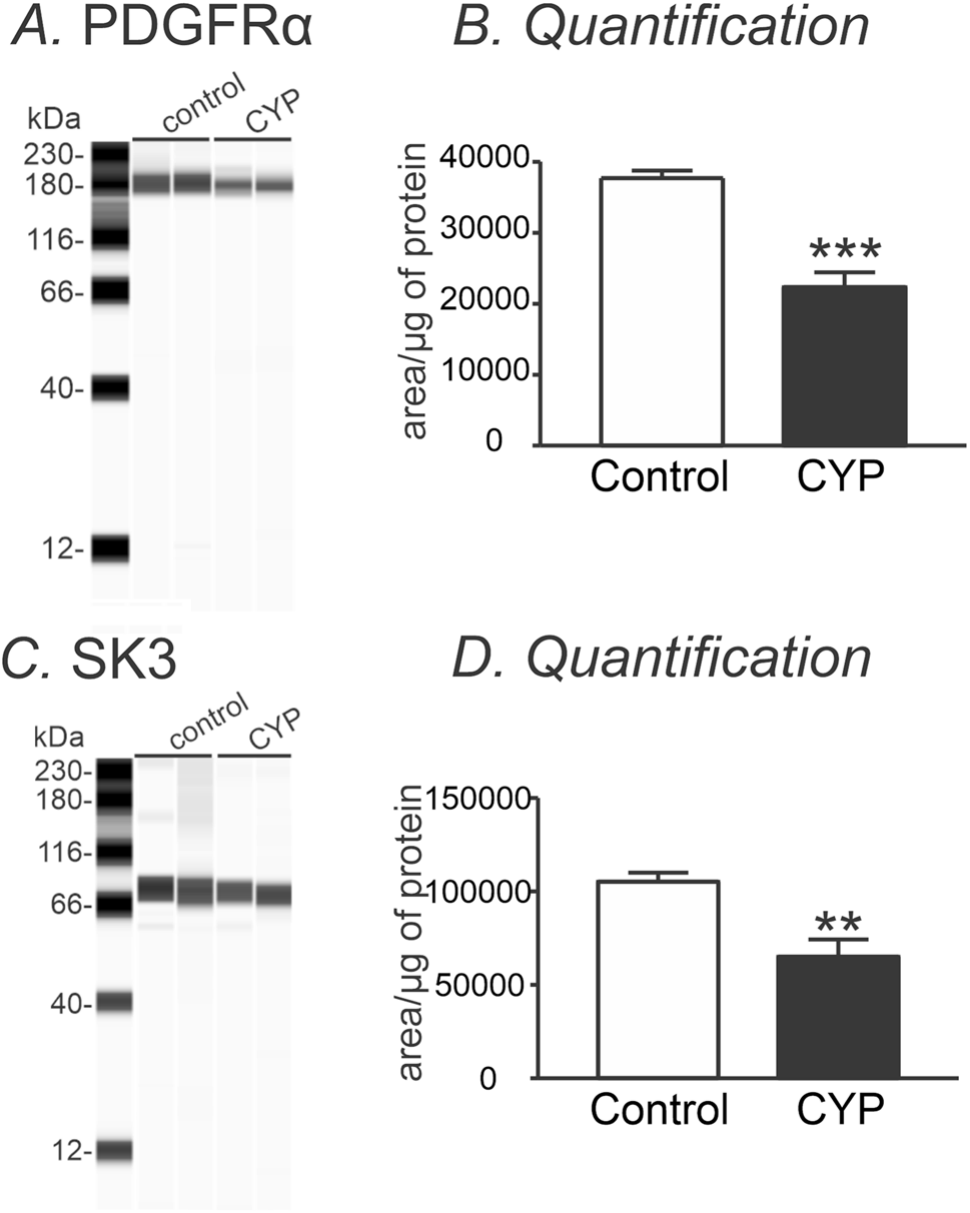
Expression levels of PDGFRα and SK3 in control and CYP-treated detrusor muscles. (**A,C**) Representative gels of PDGFRα (**A**) and SK3 (**C**) expression in control and CYP-treated detrusor muscle [Original Wes image blots for PDGFRα and SK3 (Supplementary Material)]. (**B,D**) Quantification analysis of expression level of PDGFRα (**B**) and SK3 (**D**) in control and CYP-treated detrusor muscle. **Denotes *P* < 0.005, ***denotes *P* < 0.001.

Levels of transcripts do not necessarily translate linearly into protein expression. Therefore, we also employed immunohistochemistry and Wes analysis to confirm parallel changes in protein expression. Immunohistochemistry revealed PDGFRα immune-positive cells were down-regulated in CYP-treated detrusor compared with age-matched saline-injected controls (Fig. 2A,B). Figure 2C shows a negative control image in which the primary antibody was omitted and the muscles were treated with only secondary antibody. Wes analysis showed in a more quantitative way that PDGFRα and SK3 were significantly downregulated in CYP-treated detrusor muscles, as compared to controls (n = 4, Fig. 3). These data are consistent with transcriptional data, and suggested the possibility that down-regulation of *Pdgfrα* and/or *Kcnn3* transcripts could be involved in generation of DO.

### The effect of SK channel activator on the generation of outward currents and membrane potentials in detrusor PDGFRα^+^ cells from CYP-treated bladders

**Figure 4:**
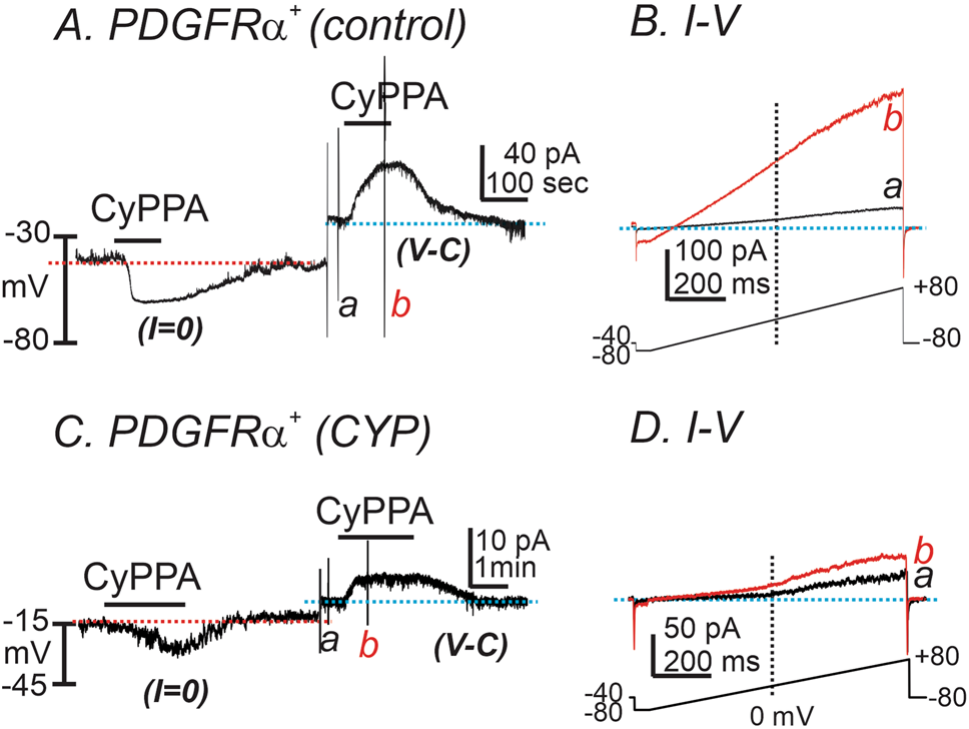
The effect of SK channel activator on membrane currents and potential in PDGFRα^+^ cells from control and CYP-treated mice. (**A**) In detrusor PDGFRα^+^ cells from control bladders, CyPPA (10 μM) induced membrane hyperpolarization under current clamp (*I* = 0, red dot line). In the same cell, CyPPA activated outward current (current above blue dotted line) at a holding potential of − 40 mV under voltage-clamp mode (V-C). (**B**) Current responses to ramp-depolarizations from (**A**) before (a) and during (b) CyPPA. Inset denotes voltage-protocol. (**C**) In detrusor PDGFRα^+^ cells from CYP-treated bladders, CyPPA (10 μM) induced membrane hyperpolarization under current clamp (*I* = 0, red dot line). In the same cell, CyPPA activated outward current (current above blue dotted line) at a holding potential of − 40 mV under voltage-clamp mode (V-C). (**D**) Current responses to ramp-depolarizations from (**C**) before (a) and during (b) CyPPA. Inset denotes voltage-protocol.

We tested the effect of a SK channel activator (CyPPA, 10 μM) on freshly dispersed detrusor PDGFRα^+^ cells. The cells were dialyzed with K^+^-rich solution (see “Methods” section). CyPPA hyperpolarized control detrusor PDGFRα^+^ cells from − 31.4 ± 4.6 to − 61.6 ± 2.9 mV under current clamp (*I* = 0, red dot line, n = 5, Fig. 4A). In the same cells, CyPPA activated outward current at a holding potential of − 40 mV in voltage-clamp (*V-C*) mode (Fig. 4A, blue dotted line). CyPPA-activated current amplitude averaged 39.3 ± 7.1 pA. Figure 4B shows currents evoked by ramp depolarization before (Fig. 4Ba, black trace) and in the presence of CyPPA (Fig. 4Bb, red trace). The resting membrane potentials (RMP) of CYP-treated detrusor PDGFRα^+^ cells were depolarized to − 16 ± 1.4 mV (n = 5, *P* < 0.01, as compared to untreated control PDGFRα^+^ cells). CyPPA induced less hyperpolarization in CYP-treated detrusor PDGFRα^+^ cells (∆mV 9.1 ± 2.0), as compared to untreated control PDGFRα^+^ cells (n = 5, *P* < 0.01, Fig. 4C) under current-clamp mode (*I* = *0*) and generated smaller outward currents under *V-C* mode (5.2 ± 0.9 pA; n = 5, *P* < 0.01, Fig. 4D) at − 40 mV. These data suggest that SK current density was significantly decreased in PDGFRα^+^ cells isolated from CYP-treated bladders, as compared with current density in control PDGFRα^+^ cells isolated from non-treated bladders.

### The effect of SK channel blocker on detrusor muscle contractions in CYP-treated bladders

**Figure 5:**
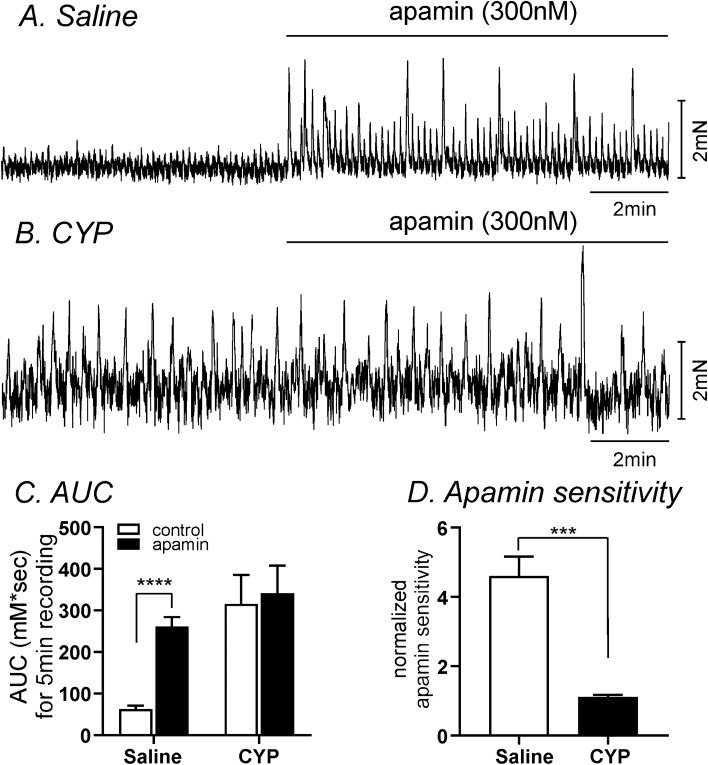
The effect of SK channel blocker on contractions in saline- and CYP-treated detrusor muscle strips. (**A,B**) Apamin increased contractility in saline-injected detrusor muscle (**A**), but did not show significant effect in CYP-treated muscle strip (**B**). (**C**) Summarized the area under the curve for 5 min recordings before (control) and after apamin in saline-injected (n = 8) and CYP-treated (n = 6) detrusor muscles. (**D**) Normalized apamin sensitivity from (**C**) in saline and CYP treated detrusor muscles. ****Denotes *P* < 0.0001 by paired t-test in (**C**) and ***denotes *P* < 0.001 by unpaired t-test in (**D**).

We examined the effect of an SK channel blocker to compare changes in functional expression of SK channels between saline-treated (control) and CYP-treated bladders using isometric force measurements. Detrusor muscle strips without submucosa exhibited spontaneous contractions. In saline-injected control, apamin (300 nM, a selective blocker of SK channels) dramatically increased AUC from 62.9 ± 7.9 to 261.5 ± 22.5 mN s during 5 min recordings (n = 8, *P* < 0.0001, Fig. 5A,C). These data are consistent with previous reports^30–32^. In CYP-treated detrusor muscle strips, spontaneous contractions were of high amplitude and irregular. AUC for these contractions was calculated since averaging the frequency and amplitude of these irregular contractions are not reliable measurements. AUC in CYP-treated muscles before and after apamin were 315.7 ± 69.8 mN s and 341.3 ± 66.4 mN s (n = 6, Fig. 5B,D), respectively. Thus, apamin had no significant effect on spontaneous contractile activity in CYP-treated muscles. We also calculated the apamin-sensitive contractions by normalized the effect of apamin from control AUC (before apamin). The sensitivity to apamin was significantly decreased in CYP-treated detrusor muscles (1.1 ± 0.1-fold) compared to saline-injected detrusor muscle strips (4.6 ± 0.6 fold, *P* < 0.001, Fig. 5D).

### The effect of SK channel blocker and agonist on CYP-treated bladders using ex vivo preparation

**Figure 6:**
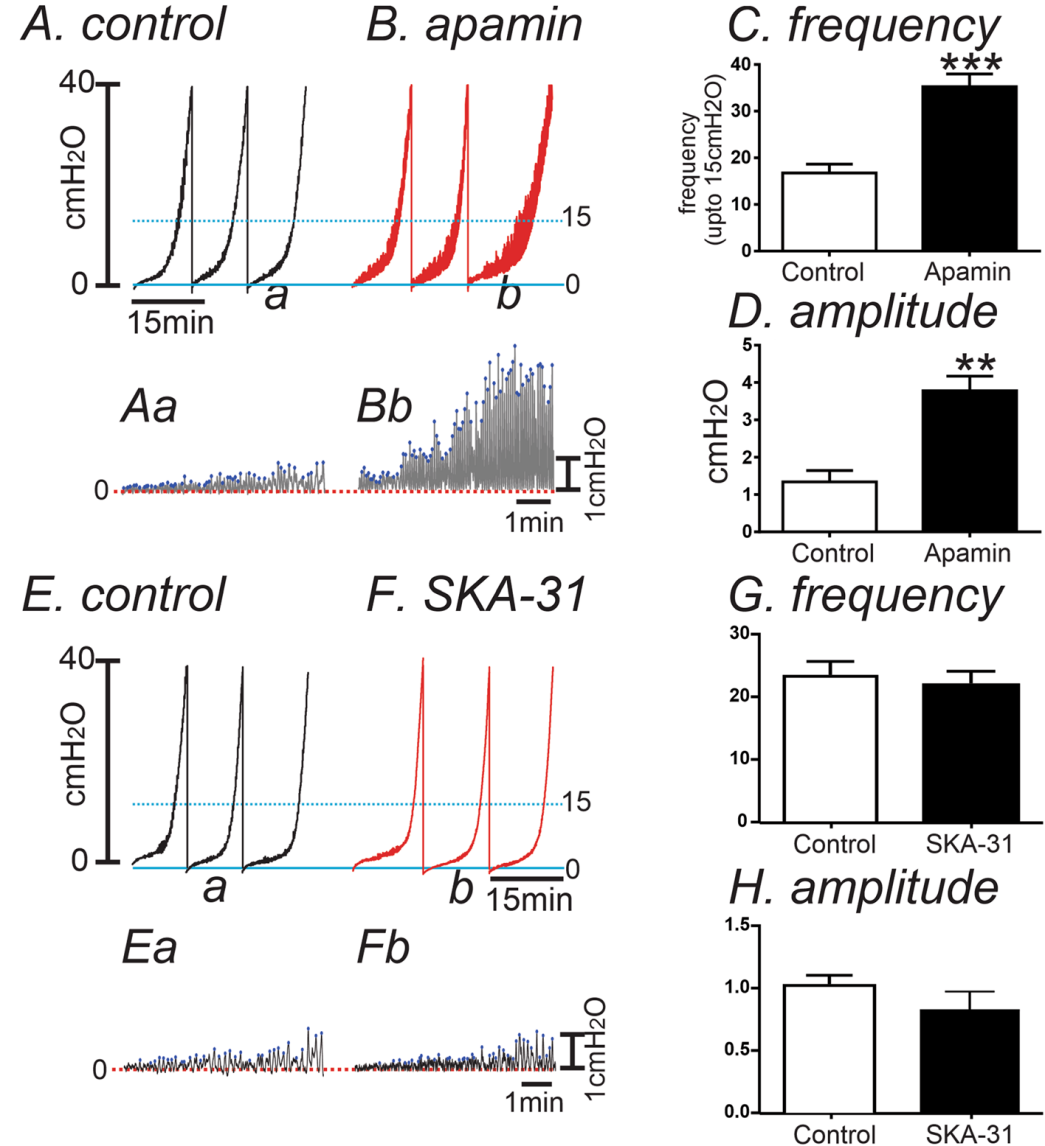
The Effect of SK channel blocker on spontaneous activity using the ex vivo volume-pressure relationship in control bladders. (**A,B**) Ex vivo compliance curves for control (**A**) and after apamin application (**B**) from control bladders up to 15 cmH_2_O (see blue dotted line). Expanded time scales with adjustment of baseline under control (**Aa**) and apamin (**Bb**) from panel (**A,B**) respectively. Blue dots were detected by threshold (0.2cmH_2_O) and used for frequency analysis. (**C,D**) Summarized frequency (**C**) and amplitude (**D**) under control and after apamin application (n = 6). ** and ***Denote *P* < 0.01 and *P* < 0.001, respectively. (**E,F**) The effect of SK chanel agonist, SKA-31 (10 µM) on control bladders without (**E**) and with SKA-31 (**F**) in ex vivo preparation. Expanded time scales with adjustment of baseline under control (**Ea**) and SKA-31 (**Fb**) from (**E,F**)***,*** respectively. (**G,H**) Summarized frequency (**G**) and amplitude (**H**) under control and after SKA application (n = 7).

**Figure 7:**
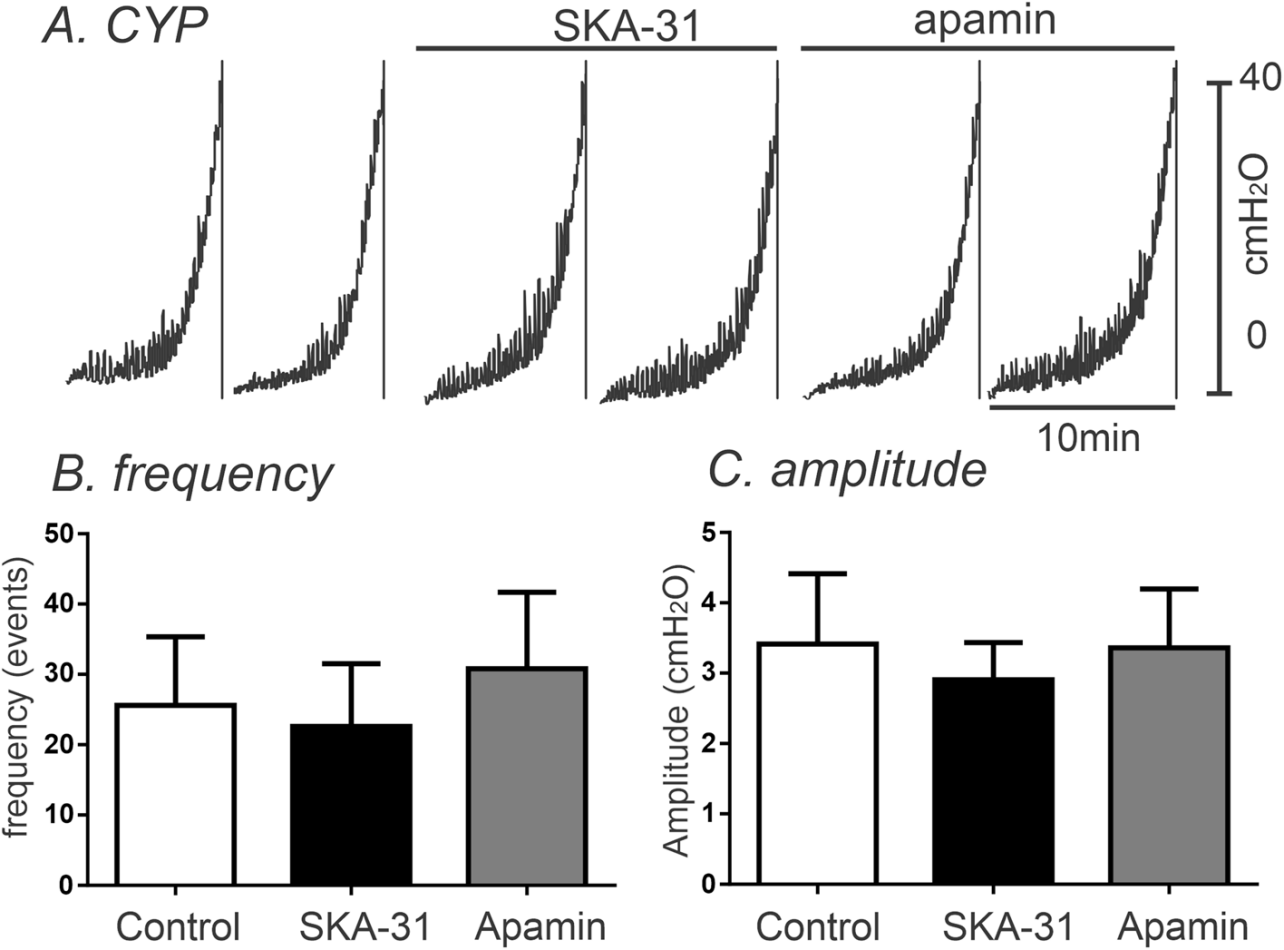
The Effect of SK channel activator and blocker on TCs in the ex vivo volume-preparation from CYP-treated bladders. (**A**) Ex vivo compliance curves for control, SKA-31 and apamin application from CYP-treated bladders. (**B,C**) Summarized frequency (**B**) and amplitude (**C**) under control, SKA31 and apamin application (n = 6) from CYP-treated bladders.

We also examined the pressure–volume relationships of excised bladders in ex vivo preparations to investigate changes in functional expression of SK channel in control and CYP-treated bladders. Ex vivo pressure–volume measurements exclude extrinsic neural regulation during filling, so this technique highlights regulation via myogenic mechanisms. In in vivo cystometry, voiding contractions start around 20 cmH_2_O in murine bladder^33^. Thus, we analyzed the pressure amplitude and frequency up to 15 cmH_2_O (see Fig. 6A,B) to associate contractile activity (i.e. transient contractions or TCs) to the non-voiding contractions (NVCs) observed in in vivo cystometry. The passive pressure underlying was normalized to 0 cmH_2_O (see Fig. 6Aa,Bb). In control bladders, infusion of KRB solution (15 μl/min) induced a small increase in intravesical amplitude (1.3 ± 0.3 cmH_2_O) with the frequency of TCs equal to 17 ± 2 events at intravesical pressures up to 15 cmH_2_O. Addition of apamin (300 nM) into the bath increased TC frequency to 35 ± 3 events (*P* < 0.001, Fig. 6A–C) and the amplitude of TCs to 3.8 ± 0.4 cmH_2_O (n = 6, *P* < 0.01, Fig. 6A,B,D). We also tested the effect of SK chanel agonist, SKA-31 on control bladders. SKA-31( 10 µM) showed negligible effect on the frequency and amplitude of TCs (n = 7, Fig. 6E–H) because SK channels may be already activated during filling. Thus, there is minmal activation by SK channel opener. CYP-treated bladders displayed increased amplitude of TCs with high frequency before apamin treatment (Fig. 7A–C). SKA-31 and apamin treatment did not significantly affect the amplitude or frequency of TCs in CYP-treated bladders (n = 6, Fig. 7B,C).

## Discussion

This study investigated the mechanisms of DO in CYP-induced cystitis. Gene transcripts and protein levels of PDGFRα and SK3 were depressed in CYP-treated detrusor muscles and in PDGFRα^+^ cells sorted to purity by FACS. The current density from SK channels evoked by a SK channel agonist, CyPPA, was also depressed in PDGFRα^+^ cells isolated from CYP-treated detrusor muscles, suggesting that gene and protein expression levels have functional consequences on bladder function. Contractile experiments supported the idea that apamin-sensitive contractions were decreased in CYP-treated detrusor muscle strips. In ex vivo experiments, an SK channel antagonist, apamin, increased the amplitude and frequency of TCs during filling in control bladders. However, the effects of apamin were reduced significantly in CYP-treated bladders. Similarly the effects of an SK agonist were also minimal in CYP-treated bladders. These data suggest that loss of PDGFRα^+^ cells and the major functional conductance provided by SK channels in these cells results in increased TCs and induction of DO in CYP-treated bladders.

Chemical cystitis is one of the adverse effects observed after administration of CYP chemotherapy in humans^34^. CYP also induces cystitis in mice and rats^13,35^. Therefore, CYP treatments of rodents has been used widely as an experimental model of IC/BPS. Single CYP intraperitoneal injection leads to urinary bladder inflammation^36^, visceral pain^8^ and DO^23^. However, the mechanisms of DO induced by CYP have not been clarified by previous studies. In the current study we tested the hypothesis that functional causes of DO after CYP treatment could be related to loss-of-function of bladder regulation provided by PDGFRα^+^ cells.

Previous studies have shown that as the bladder fills, volume increases and the walls are stretched, but intravesical pressure remains low during much of the period of filling^37^. This accommodation occurs even though there is a natural tendency for detrusor SMCs to contract in response to stretch^38,39^. During bladder filling, NVCs are detected in cystometric records from various species^40–43^. NVCs appear to correspond to localized contractions that are also observed in ex vivo bladder preparations and have been termed ‘spontaneous phasic contractions’, ‘micromotions’ or ‘TCs’^44–48^. TCs increase as bladder filling proceeds^44^. Activation of non-selective cation channels expressed in detrusor SMCs^38,39^ generates TCs which initiate sensory inputs via activation of afferent nerves^44^ that propagate to the central nervous system during bladder filling^49–52^. However, under physiological condition, development of TCs can be restrained due to activation of SK channels in detrusor muscles^26^. For instance, SK channel activators reduce and SK channel blocker increase detrusor muscle contractions^30,32,53–55^. Furthermore, *Kcnn3* knockout mice show an increase in NVCs in in vivo cystometry and TCs in ex vivo bladders^44,56^. Previous reports demonstrate that *Kcnn3* are highly expressed in detrusor PDGFRα^+^ cells^26,29,57^. The current density of SK channels is very low in SMCs compared to detrusor PDGFRα^+^ cells^25^^.^ Although there is no direct evidence of electrical coupling between PDGFRα^+^ cells and SMCs, it seems clear that PDGFRα^+^ cells may serve an important stabilizing role in regulating contractile activity of SMCs during bladder filling. Therefore, disruption of PDGFRα^+^ cells and/or downregulation of SK channels and intracellular signaling pathways regulating SK channel activity could lead to DO.

In the present study we found that *Kcnn3* and *Pdgfra* transcripts were depressed in CYP-treated detrusor muscles and in detrusor PDGFRα^+^ cells from CYP-treated PDGFRα/eGP mice. In contrast, *Kcnma1* and *Cacna1C* that encode important proteins that have been implicated in the regulation of bladder excitability were unchanged from control SMCs in mice treated with CYP. Western analysis confirmed that both PDGFRα and SK3 proteins were reduced in CYP-treated detrusor muscles.

Immunohistochemistry also showed a reduction in the density of PDGFRα^+^ cells in CYP-injected detrusor muscle. At present we do not know what factors, activated by CYP, might be responsible for loss of PDGFRα^+^ cells and downregulation of SK channels in detrusor muscles, however the upregulation of inflammatory factors, such as *Tnfα* and *Il6*, suggest that an inflammatory mechanism may be involved.

Functional studies to evaluate the state of the SK conductance in detrusor PDGFRα^+^ cells before and after CYP treatment were performed using the patch clamp technique. Our recordings confirmed the presence of an SK conductance in control (untreated) PDGFRα^+^ cells, and the availability of this conductance was decreased in PDGFRα^+^ cells isolated from CYP-treated bladders. As above, CYP treatment led to reduced PDGFRα^+^ cell density, and the patch clamp experiments showed that there was concomitant reduction in the SK conductance normally prominent in these cells. Since SK channels provide stabilization of membrane potential and excitability of SMCs during bladder filling, reducing the availability of the SK conductance would lead to increased generation of TCs. This hypothesis was confirmed using isometric force measurments of detrusor muscle strips and ex vivo bladder preparations of mice treated with CYP. We found an increase in TCs as the bladders were filled and less sensitivity of apamin in comparison to the control mice.

In conclusion, we found that CYP treatments induced DO was caused by reduced detrusor PDGFRα^+^ cells and reduction in the prominent SK conductance expressed by these cells that is utilized to regulate SMC excitability during bladder filling. These experiments provide a novel understanding of detrusor PDGFRα^+^ cells and how defects in these cells can contribute to the development of abnormal bladder activity.

## Methods

### Preparation of tissue

Male C57BL/6J, *Pdgfra*^*tm11(EGFP)Sor*^/J (PDGFRα/eGFP) and smMHC/Cre/eGFP were purchased from Jackson Laboratory, Bar Harbor, ME. The mice were maintained and experiments were carried out in accordance with the National Institutes of Health Guide for the Care and Use of Laboratory Animals. All methods are reported in accordance with ARRIVE guidelines. Animal protocols were approved by the University of Nevada, Reno Institutional Animal Care and Use Committee. Mice were housed in a pathogen-free barrier facility on a 12-h light/dark cycle with free access to water and food (Prolab 5P76 Isopro 3000; 5.4% fat by weight). All mice were males and used at 8–10 weeks of age for all experiments (purchased from Jackson Laboratory, Bar Harbor, ME, USA). Mice were sacrificed with isoflurane inhalation (AErrane; Baxter, Deerfield, IL, USA) followed by cervical dislocation. The abdomens were opened and bladders were removed, then placed in Krebs–Ringer Bicarbonate (KRB) buffer solution (see below). The bladders were opened and the urothelium and detrusor layer were isolated by sharp dissection^26^.

### Induction of CYP-induced cystitis

Murine CYP-induced cystitis was established according to previously described protocols^23,36,58^. To induce acute cystitis, C57BL/6J, *Pdgfra*^*tm11(EGFP)Sor*^/J (PDGFRα/eGFP) and smMHC/Cre/eGFP were injected with 1.2 mg of CYP per 100 μl of saline solution and control mice were injected only with saline solution. Both mice were sacrificed on day 7 after CYP treatment.

### RNA isolation, reverse-transcription PCR and quantitative PCR

For quantitative analysis of transcripts, PDGFRα^+^ cells and SMCs purified by fluorescence‐activated cell sorting (FACS), and detrusor muscles were used for molecular tests as previously described^26^. Total RNA was isolated from the detrusor smooth muscle tissues and sorted cells using Direct-zol RNA miniPrep Kit (Zymo Research, Irvine, CA, USA), and first-strand cDNA was synthesized using qScriptTM cDNA SuperMix (Quanta, Gaithersburg, MD, USA) according to the manufacturer’s instructions. Endpoint PCR was performed with specific primers (Table 1) using Go-Taq Green Master Mix (Promega Corp., Madison, WI, USA). Products of the end-point PCR were run on a 2% agarose gel and visualized by ethidium bromide. Next, the standard curve method of Quantitative PCR (qPCR) was performed as previously described in Bookout et al. 2005 with the same primers as PCR. The fast SYBR Green Master Mix (Life Technologies, Grand Island, NY, USA) on the Quantstudio 3 Real Time PCR System (Applied Biosystems) was also employed. In summary, mean values of duplicate samples for the diluted cDNA had regression analysis performed on them and this was used to generate standard curves. This resulted in transcriptional quantification of each gene, log transformation of corresponding raw data was taken and transcription expression was given relative to the endogenous glyceraldehyde 3-phosphate dehydrogenase (*Gapdh*).

**Table 1:** Primer sequences used for qPCR.

Gene name	Primer sequences	Accession number
*Gapdh*	F-GCCGATGCCCCCATGTTTGTGA R-GGGTGGCAGTGATGGCATGGAC	NM_008084.3
*Pdgfra*	F-ATGACAGGAGGGAGGGCTTCAACG R-CGGCACAGGTCACCACGATCGTTT	NM_011058.2
*Tnf*	F-CTGAACTTCGGGGTGATCGG R-GGCTTGTCACTCGAATTTTGAGA	NM_013693.3
*Il6*	F-TCCAGTTGCCTTCTTGGGAC R-GTACTCCAGAAGACCAGAGG	NM_031168.2
*Cacna1c*	F-GTAAGGATGAGTGAAGAAGCCGAGTAC R-CAGAGCGAAGGAAACTCCTCTTTGG	NM_009781
*Kcnma1*	F-GGTGATCTGTTCTGCAAAGCTCTG R-GTTGGTACGAGCTCAAACTCGTAG	NM_001253358
*Kcnn1*	F-TGTGTTGTTGGTCTTCAGCG* R-ACACACCCTTCCCACAGTAG	NM_032397.2
*Kcnn2*	F-TTCTAACAACCTGGCGCTCT R-CCAGCTTGTAGCCGATGTTC	NM_080465.2
*Kcnn3*	F-CTGCTGGTGTTCAGCATCTCTCTG R-GTCCCCATAGCCAATGGAAAGGAAC	NM_080466.2
*Kcnn4*	F-AAGATGCTGGCCGCCATCCACA R-TCTTCTCCAGGGCACGGTGCGA	NM_008433.4

### Immunohistochemistry

Whole bladder was fixed in paraformaldehyde [4% w/v in 0.1 m phosphate buffer solution (PBS) for 60 min at 4 °C] and washed with PBS. Fixed tissue was passed through sucrose gradient (up to 30%). Tissues were bisected and snap frozen on liquid nitrogen in Tissue Tek OCT compound (Sakura Finetek, USA). Ten micron cryosections were cut on a cryostat (Leica CM3050) and placed on to Vectabond (Vector Labs, USA) coated slides. Tissues were washed 5 times and incubated in BSA (1%) for 1 h at room temperature containing Triton X‐100 (0.3% in PBS) to reduce non‐specific antibody binding. Tissue sections were incubated overnight in primary anti PDGFRα antibody (R&D Systems, 1:100 dilution) diluted in 0.5% Triton X-100 at 4 °C. Excess primary antibody was washed in PBS and were then incubated Alexa Fluor 488 (Invitrogen, Grand Island, NY, USA) secondary antibody diluted 1:1000 in PBS for 1 h. Excess secondary antibody was washed in PBS and mounted with Aqua mount mounting media (Lerner Laboratories, Pittsburgh, PA, USA). Secondary antibody only was used for negative control to examine autofluorescence or non-specific staining. Sections on glass slides and cover slipped were imaged with the Olympus FV1000 (Olympus America Inc., Center Valley, PA, USA) and Carl Zeiss LSM 510 confocal microscope (Carl Zeiss Microimaging, LLC, Thornwood, NY, USA). Adobe Photoshop CS5 (Adobe Systems Incorporated, San Jose, CA, USA) was used to arrange the images taken.

### Wes Simple Western automated capillary electrophoresis and immunodetection

Tissue samples were prepared by homogenizing at 4 °C in 0.3 ml radioimmune precipitation assay (RIPA) buffer (1×/type) with added protease inhibitor tablet (Thermo-Fisher mini tablets EDTA free) with a Bullet Blender (5 min, speed 5, 1 stainless steel bead per detrusor muscle). The homogenate was centrifuged at 4 °C, 3000×*g* for 10 min, to remove cell debris. The supernatant was aliquoted and stored at − 80 °C^59^. Other tissue samples were subjected to differential centrifugation to obtain a plasma membrane-enriched fraction for PDGFRα and SK3 detection. In this case, the tissues were homogenized in ice cold lysis buffer (mM; 50 Tris HCl pH 8.0, 60 beta-glycerophosphate, 100 NaF, 2 EGTA, 25 Na-pyrophosphate, 1 DTT, and protease inhibitor tablet). Each tissue was homogenized in 0.3 ml lysis buffer, centrifuged at 16,000×*g* at 4 °C for 10 min, and the supernatants centrifuged at 100,000×*g* for 1 h at 4 °C. The 100,000×*g* pellet was resuspended into 0.3 ml of lysis buffer, and stored at − 80 °C. Protein concentrations of the supernatants were determined by the Bradford assay using bovine γ‐globulin as the standard. Automated western blotting (Wes Simple Western, ProteinSimple, Santa Clara, CA) was utilized to measure PDGFRα and SK3 protein levels. Simple Western analysis was performed according to the ProteinSimple user manual. The primary antibodies and total protein lysate concentrations for each protein were determined by initial titrations of lysate amounts and antibody dilutions. Final concentrations of bladder lysates were 1.0 mg/ml and 0.3 mg/ml for SK3 and PDGFRα respectively. The antibody for SK3 (catalog #sc-28621, Santa Cruz Biotechnologies, CA, USA) was used at a1:100 dilution. The antibody for PDGFRα (catalog #sc-338, Santa Cruz Biotechnologies, CA, USA) was used at a 1:100 dilution. The boiled samples, biotinylated protein ladder, blocking buffer, primary antibodies, secondary antibodies, chemiluminescent substrate, and wash buffer were loaded into the plate (Wes 12–230 kDa Pre-filled Plates with Split Buffer, ProteinSimple). The plate was then loaded onto the automatic size-based Simple Western system for protein separation, antibody incubation and imaging using the Wes default parameters. Image reconstruction of the detected proteins was generated by Compass software (ProteinSimple). The protein signals were quantified from the eletropherogram of the area under the chemiluminescent intensity peak obtained by Compass software.

### Electrophysiological recordings

Whole cell currents and membrane potentials were recorded using whole cell voltage- and current-clamp techniques. Cells were placed in a 0.5 ml chamber mounted on an inverted microscope (Nikon Eclipse Ti-E, Japan) equipped with fluorescence objective (40×, Nikon CFI Flour objective). This microscope was equipped with Xenon arc illumination with a GFP filter set to visualize cells expressing fluorescent reporters. PDGFRα^+^ cells, isolated from *Pdgfra*^*tm11(EGFP)Sor*^/J mice, were identified by the fluorescence of eGFP in nuclei under. Pipette tip resistances were: 4–6 MΩ for PDGFRα^+^ cells. An Axopatch 200B amplifier with a CV-4 headstage (Molecular Devices, Sunnyvale, CA, USA) was used. All data were analysed using pCLAMP software (Axon Instruments, USA) and Graphpad Prism (v. 3.0, Graphpad Software Inc., SanDiego, CA, USA). All recordings were made at room temperature of ∼ 21 °C.

### Isometric force measurements

Tension experiments were performed using standard organ bath techniques to measure the changes in force. The bladders were cut from the neck to the base. The urothelium was peeled off and the bladder was cut into 4 equal longitudinal strips of 1.5 × 5 mm. One end of the strip was attached to a fix mount and the other to a force transducer (Grass FT03, Grass Instrument Co.). Muscles were immersed in organ baths perfused with oxygenated (95% O2 and 5% CO2) KRB solution. The bath temperature was maintained at 37.5 ± 0.5 °C. A resting force of 1 g was applied and the bladder strips were left to equilibrate from 1 to 2 h. Mechanical responses were recorded on a computer running LabChart (ADInstruments, Colorado Springs, CO, USA). The area under the curve (AUC) was calculated by Clampfit (version 10.1, Molecular Devices, Sunnydale, CA, USA) after adjustment baseline to measure only active contractions.

### Ex vivo preparation

This technique is used to establish the relationship between storage volume and pressure. The bladders were extracted and the urethras ligated, close to the vesico-ureteric junctions. A catheter was placed in the urethral opening to record pressure and filling. Intravesical pressure was recorded in reference to atmospheric pressure. An amplifier was connected to a transducer that has been filled with water. A syringe filled with KRB solution was connected to a pump. The infusion rate of the KRB solution was 15–25 µl/min by automatic infusion and this was kept at 37 °C. The filling of the bladder with KRB solution was stopped when the pressure reaches 45–50 cmH_2_O. This is to avoid distending the bladder which can cause permanent tissue damage. Recordings obtained were analyzed by Clampfit (Molecular device) which had the baseline adjusted to examine the amplitude and frequency of pressures during filling (see Fig. 5).

### Solutions and chemicals

Whole-cell configuration was achieved in Ca^2+^-containing physiological saline bath solution (mm): NaCl 135, KCl 5, MgCl_2_ 1.2, CaCl_2_ 2, glucose 10, Hepes 10, pH 7.4 with Tris-base. The pipette solution contained (mm): KCl 135, CaCl_2_ 0.012, MgATP 3, Na_2_GTP 0.1, creatine phosphate disodium 2.5, EGTA 0.1, glucose 10, Hepes 10, pH 7.2 with Tris-base. The KRB buffer solution contained (mM): NaCl 120, KCl 5, CaCl_2_ 2, MgCl_2_ 1 NaHCO3 25, d-glucose 5.5. All drugs and reagents including apamin and CyPPA (*N*-cyclohexyl-*N*-[2-(3,5-dimethyl-pyrazol-1-yl)-6-methyl-4-pyrimidinamine) were purchased from Sigma.

### Statistical analyses

All data were expressed as means ± SEM. “n” denotes the number of animals used. All statistical analyses were performed using Graphpad Prism. Student’s paired or non-paired *t* test were used to compare groups of data and differences were considered to be significant at *P* < 0.05. Data analysis for the westerns was performed using Compass software (ProteinSimple, San Jose, CA, USA), and expressed as intensity area/ug of protein. Lane view images of the western blots were saved as JPEGs, opened with Adobe Photoshop, converted to TIFFs; and saved after adjusting the image resolution with the Auto Res function.

## Supplementary Information

Supplementary Figure 1.

## Data Availability

The datasets used and/or analyzed during the current study available from the corresponding author on reasonable request. All data generated or analyzed during this study are included in this published article.
