# Supplementary figures and images for: Role of detrusor PDGFRα+ cells in mouse model of cyclophosphamide-induced detrusor overactivity
Source: Sci Rep. 2022 Mar 24;12:5071. doi: 10.1038/s41598-022-09155-3 (PMC8948241; doi:10.1038/s41598-022-09155-3)

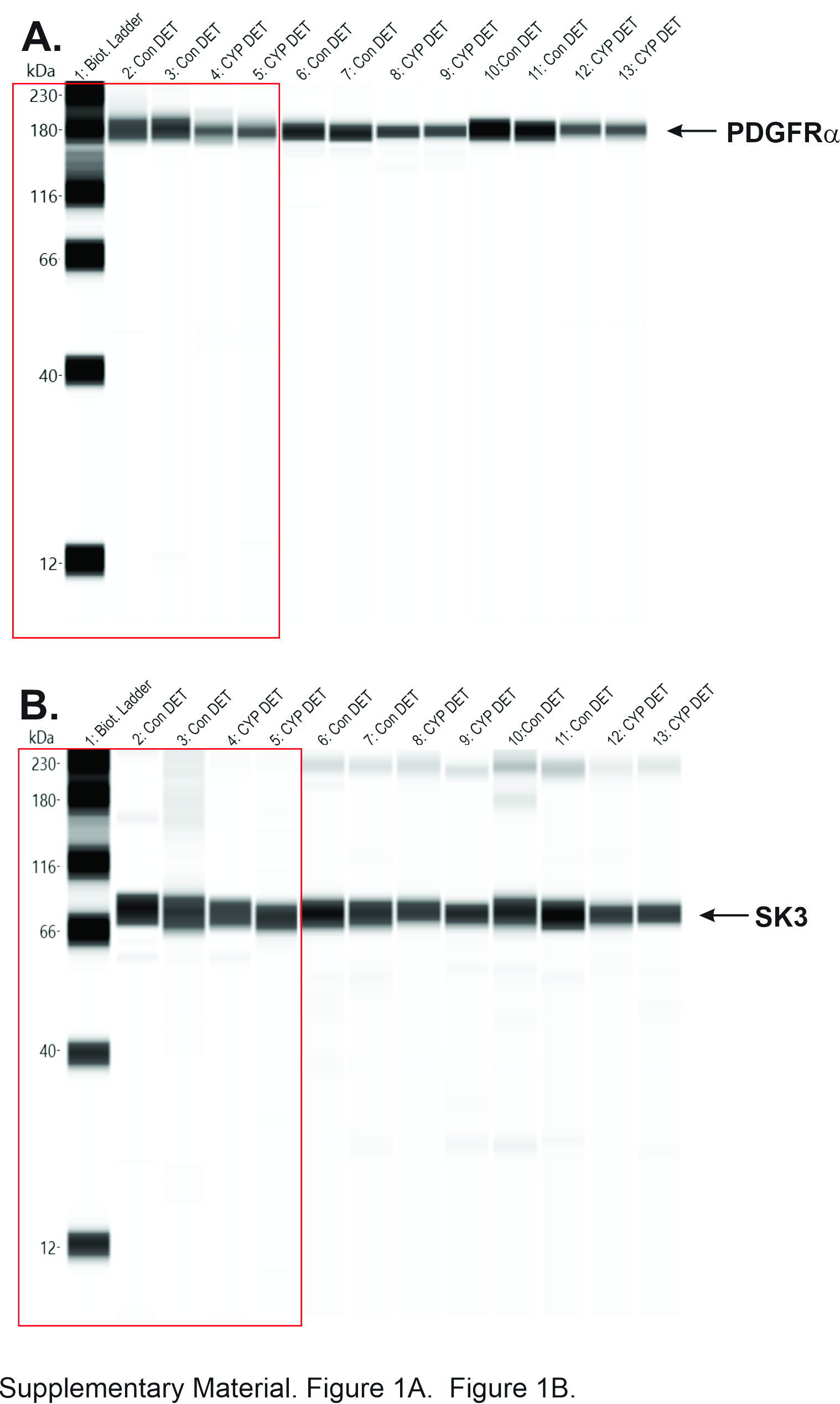

Supplement: Supplementary file 1 — Supplementary Figure 1. [file 41598_2022_9155_MOESM1_ESM.tif]
